# Discovery of powdery mildew resistance gene candidates from *Aegilops biuncialis* chromosome 2M^b^ based on transcriptome sequencing

**DOI:** 10.1371/journal.pone.0220089

**Published:** 2019-11-11

**Authors:** Huanhuan Li, Zhenjie Dong, Chao Ma, Xiubin Tian, Zhiguo Xiang, Qing Xia, Pengtao Ma, Wenxuan Liu

**Affiliations:** 1 College of Life Sciences, Henan Agricultural University, Zhengzhou, Henan Province, China; 2 Wheat Research Center, Henan Academy of Agricultural Sciences, Zhengzhou, Henan Province, China; 3 College of Life Sciences, Yantai University, Yantai, Shandong Province, China; Institute of Genetics and Developmental Biology Chinese Academy of Sciences, CHINA

## Abstract

Powdery mildew is one of the most widespread diseases of wheat. The development and deployment of resistant varieties are one of the most economical and effective methods to manage this disease. Our previous study showed that the gene(s) at 2M^b^ in Chinese Spring (CS)-*Aegilops biuncialis* 2M^b^ disomic addition line TA7733 conferred a high level of resistance to powdery mildew of wheat. In this study, resistance spectrum of TA7733 was assayed by using 15 *Blumeria graminis* f. sp. *tritici* (*Bgt*) isolates prevalent in different regions of China. The result indicated that TA7733 was highly resistant to all tested *Bgt* isolates and the gene(s) on chromosome 2M^b^ conferred broad-spectrum resistance to powdery mildew. In order to characterize mechanism of powdery mildew resistance by identifying candidates R-genes derived from *Ae*. *biuncialis* chromosome 2M^b^ and develop 2M^b^-specific molecular markers, we performed RNA-seq analysis on TA7733 and CS. In total we identified 7,278 unigenes that showed specific expression in TA7733 pre and post *Bgt*-infection when compared to CS. Of these 7,278 unigenes, 295 were annotated as putative resistance (R) genes. Comparatively analysis of R-gene sequences from TA7733 and CS and integration CS Ref Seq v1.0 were used to develop R-gene specific primers. Of 295 R-genes we identified 53 R-genes were specific to 2M^b^ and could be involved in powdery mildew resistance. Functional annotation of majority of the 53 R-genes encoded nucleotide binding leucine rich repeat (NLR) protein. The broad-spectrum resistance to powdery mildew in TA7733 and availability of 2M^b^-derived putative candidate R-gene specific molecular markers identified in this study will lay foundations for transferring powdery mildew resistance from 2M^b^ to common wheat by inducing CS-*Ae*. *biuncialis* homoeologous recombination. Our study also provides useful candidates for further isolation and cloning of powdery mildew resistance gene(s) from *Ae*. *biunciali*s chromosome 2M^b^.

## Introduction

Common wheat (*Triticum aestivum* L., 2*n* = 6*x* = 42, AABBDD), one of the most widely planted crops in the world provides 20% of the calories and 25% of its protein consumed by human [1,2]. Wheat production plays an important role in food security and social stabilization. However, wheat yields and quality are severely threatened by various diseases, such as rusts, *Fusarium* head blight (FHB) and powdery mildew. Wheat powdery mildew, caused by *Blumeria graminis* f. sp. *tritici* (*Bgt*), is one of the most destructive diseases all over the world, with severe yield losses ranging from 13% to 50% [3,4]. In recent years, certain agronomic practices to increase yields, such as popularization of high planting density, high inputs of irrigation and fertilization have accelerated the spread and severity of powdery mildew [5,6]. Though spraying fungicides can reduce the damage caused by this disease to some extent, it can also result in side effects such as drug resistant of powdery mildew fungus, environment pollution, and high production inputs [7]. Breeding disease-resistant varieties is currently recognized as one of the most effective and economical ways to control powdery mildew.

Wild relatives of common wheat contain a large number of *Bgt* resistance genes for wheat improvement. Up to now, the number of designated genes of powdery mildew resistance (*Pm*) was more than 80 at 54 loci [8,9], of which approximately half of *Pm* genes were derived from wild relatives of wheat. However, some *Pm* genes had been defeated by new virulent *Bgt* races or by races that were previously present at very low frequencies in the pathogen population [10,11], and some were difficult to use in wheat breeding because of linkage drags [12,13]. Therefore, ongoing efforts to explore and identify new powdery mildew resistance genes are needed for wheat breeding programs.

*Aegilops biuncialis* (2*n* = 4*x* = 28, U^b^U^b^M^b^M^b^) is a tetraploid wild relative of wheat, belonging to the section *Polyeides* of the genus *Aegilops*. The U^b^ genome of it was derived from *Ae*. *umbellulata* (2*n* = 2*x* = 14, UU), and M^b^ genome from the diploid *Ae*. *comosa* (2*n* = 2*x* = 14, MM) [14,15]. *Aegilops biuncialis* owns many desired agronomic traits for wheat improvement, such as resistance to yellow rust [16], brown rust [17], powdery mildew and barley yellow dwarf virus [18], tolerance to drought and salt [19–21], high micronutrients contents [22], and special high molecular weight glutenin subunits [23]. Successful attempts have been made to cross *Ae*. *biuncialis* with wheat, develop a series of wheat-*Ae*. *biuncialis* addition lines, and transfer desired genes from *Ae*. *biuncialis* into wheat [20,24,25]. In previous study, we identified that CS-*Ae*. *biuncialis* 2M^b^ disomic addition line TA7733 conferred high resistance to powdery mildew compared with its recipient parent CS [26].

The isolation and cloning of plant disease resistance genes had great significance for both plant disease resistance breeding and the study on molecular mechanisms of disease resistance. Map-based cloning is currently an important method to isolate novel genes. However, it is very challenging to perform fine mapping and map-based cloning of alien genes derived from wild relatives of wheat due to the strict control of homoeologous recombination by pairing homoeologous (*Ph*) genes in hexaploid wheat backgrounds [27–29]. Furthermore, molecular markers of alien chromosome-specificity were limited for fine mapping of alien genes. Regardless, with the rapid development of high-throughput sequencing, sequencing-based technologies such as RNA-seq have been frequently used to develop molecular markers [1,30,31], detect expression pattern and level of genes responded to pathogens [32], exploit new genes and identify gene function without prior information of the particular reference genome sequences [33,34]. RNA-seq is very helpful to explore disease-resistant genes derived from wild relatives. For example, Li et al. (2016) obtained eight powdery mildew resistance-related genes from *Thinopyrum intermedium* by RNA-seq analysis [35]. Zou et al. (2018) successfully isolated a powdery mildew resistance gene *Pm60* from *T*. *urartu* by combining genetic mapping and RNA-seq analysis [9].

In this study, we report the assays of a broad-spectrum resistance gene(s) on chromosome 2M^b^ derived from *Ae*. *biuncialis*, discovery of 2M^b^-specific candidate genes of powdery mildew resistance, and development of molecular markers of 2M^b^ specificity based on transcriptome sequencing of CS-*Ae*. *biuncialis* 2M^b^ disomic addition line TA7733. This study will provide foundations for the transfer and cloning of resistance gene(s) from chromosome 2M^b^, as well as further understanding of the molecular and genetic mechanisms of disease resistance conferred by *Ae*. *biuncialis* chromosome 2M^b^.

## Materials and methods

### Plant materials

Common wheat landrace CS (2*n* = 6*x* = 42, AABBDD), *Ae*. *comosa* TA2102 (2*n* = 2*x* = 14, MM), and CS-*Ae*. *biuncialis* 2M^b^ disomic addition line TA7733 (2*n* = 44) where a pair of 2M^b^ chromosomes derived from *Ae*. *biuncialis* were added into CS genetic background were used in this study. All the materials were kindly provided by the Wheat Genetics Resource Center at Kansas State University, USA and maintained at the experimental station of Henan Agricultural University, China.

### Cytogenetic analysis

Chromosome spreads were prepared from root tip cells as described by Huang et al. (2018) [36]. The cytological observations were performed using a BX51 Olympus phase contrast microscope (Olympus Corporation, Tokyo, Japan).

Genomic DNA (gDNA) was extracted from fresh leaves using a modified hexadecyl trimethyl ammonium bromide (CTAB) method [37]. The concentration and purity of DNA were measured with the Nanophotometer P360 (Implen GmbH, München, Germany).

Genomic *in situ* hybridization (GISH) was applied to analyze the chromosomal composition of TA7733. Genomic DNA of *Ae*. *comosa* accession TA2102 (genome M^b^ donor of *Ae*. *biuncialis*) and wheat CS were respectively used for probe labeling with fluorescein-12-dUTP and blocking at a ratio of 1:130 to distinguish *Ae*. *biuncialis* 2M^b^ chromosome. GISH was carried out as described by Liu et al. (2017) [38]. Hybridization signals were observed under an OLYMPUS AX80 (Olympus Corporation, Tokyo, Japan) fluorescence microscope, captured with a CCD camera (Diagnostic Instruments, Inc., Sterling Heights, MI, USA) and processed with Photoshop CS 3.0.

After GISH, the hybridization signals were washed off with phosphate-buffered saline (PBS). Eight single-strand oligonucleotides were then used as probes for dual-color nondenaturing fluorescence *in situ* hybridization (ND-FISH) [36,39]. The eight oligonucleotides includes Oligo-pAs1-1, Oligo-pAs1-3, Oligo-pAs1-4, Oligo-pAs1-6, Oligo-AFA-3, Oligo-AFA-4, Oligo-pSc119.2–1 and Oligo-(GAA)_10._ The first six were labeled with 6-carboxytetramethylrhodamine (TAMRA) generating red signals, and the last two being labeled with 6-carboxyfuorescein (FAM) generating green signals. All the oligonucleotides were synthesized at Sangon Biological Technology, Shanghai, China.

### Evaluation of powdery mildew resistance

A mixture of prevailing *Bgt* isolates collected in Henan Province were used to evaluate the resistance of CS and CS-*Ae*. *biuncialis* 2M^b^ disomic addition line TA7733. Fifteen prevalent *Bgt* isolates collected from different regions of China were chosen to evaluate the resistance spectrum of TA7733 at the seedling stage by using CS as a susceptible control. The 15 *Bgt* isolates were provided by Prof. Pengtao Ma, Yantai University, China. They were all single-pustule-derived powdery mildew virulent isolates by separate artificial inoculation. The infection type (IT) were scored 7–10 days post-inoculation using a 0 to 4 rating scale [40], with 0 as immune, 0; as nearly immune, 1 as highly resistant, 2 as moderately resistant, 3 as moderately susceptible, and 4 as highly susceptible. IT 0 to 2 were considered as resistance, while IT 3 to 4 were being susceptible.

At 10 days post-*Bgt* inoculation, the first leaves of TA7733 and CS were cut into 2 cm segments and stained with coomassie brilliant blue following Li et al. (2016) [35] for further microscopic observation of *Bgt* development on the leaves.

### Illumina library construction and sequencing

Seeds of CS and TA7733 soaking in water for 24 h at 23°C were transferred into a mixture of nutrient soil and vermiculite (1:1). Seedlings with full extended first leaf were dusted using fresh conidiophores of *Bgt* isolates. Leaves at 0, 12, 24, 48 and 72 hours post-inoculation (hpi) were respectively collected, rapidly frozen in liquid nitrogen and stored at -80°C for RNA extraction.

Total RNA of ten samples (0, 12, 24, 48 and 72 hpi for CS and TA7733, each) were extracted for transcriptome sequencing. Then equal amounts of RNA samples 12–72 hpi from TA7733 and CS were mixed to generate RNA-seq sample RI and SI, respectively. RNA at 0 hpi from TA7733 and CS were accordingly represented as RNA-seq sample RC and SC. Two biological replicates were performed in this study, forming a total of eight RNA samples (RI1, RI2, RC1, RC2, SI1, SI2, SC1 and SC2). The designations 1 and 2 are used to represent replicates 1 and 2, respectively. Libraries with an average insert size of 200 bp constructed from these eight samples were then sequenced using the Illumina HiSeqTM 2500 by the Beijing Genomics Institute.

### Reads processing, assembly, and sequence annotation

Prior to assembly, sequencing raw reads were pre-processed using a Perl script dynamic-Trim.pl to remove the adaptor sequences, low-quality sequences, low complexity sequences, short reads and empty reads. Reads data with a quality score (Qphred) ≥ 50 (Q50: ratio of an error rate of 0.01%) were then merged and input into the data assembly software Trinity for assembling into transcripts. The generated unigenes were annotated by a Blastx alignment search (E-value<10^−5^) against the NCBI non-redundant (NR) protein, SWISSPROT, gene ontology (GO), eukaryotic orthologous groups (KOG), kyoto encyclopedia of genes and genomes (KEGG) and plant resistance gene (PRG) databases.

### Amplification and analyses of candidate disease resistance genes

R gene-specific primer sets were designed based on their transcriptome sequences to perform PCR amplification using gDNA from TA7733 and CS as templates to verify 2M^b^ specific genes. PCR amplification were conducted in 15 μl reaction volumes containing 2 μl template gDNA (100 ng/μl), 0.25 μl forward primer (10 μmol/l), 0.25 μl reverse primer (10 μmol/l), 7.5 μl Taq MasterMix (CW Bio Inc., China) and 5 μl ddH_2_O. PCR cycling conditions were as follows: 94°C for 5 min followed by 35 cycles of 94°C for 30 s, 50–66°C for 30 s, and 72°C for 1 min, followed by a final 10-min extension at 72°C. The PCR products were digested with four base-restriction enzymes. Five microliters of a restriction enzyme mixture containing 2.8 μl of ddH_2_O, 2.0 μl of CutSmart buffer, and 0.2 μl of an enzyme stock solution was added to 15 μl of PCR products and incubated for 3.5 h at 65°C. The PCR or restricted PCR products were separated on a 2.0% agarose gel-electrophoresis stained with ethidium bromide and visualized by UV light.

### Mapping candidate disease resistance genes onto chromosome 2M^b^

Genome sequences of wheat landrace CS (CS Ref Seq v1.0) were used as references in Blastn searches to obtain position information for R genes from *Ae*. *biuncialis* chromosome 2M^b^. Comparative maps of 2M^b^-specific R genes were made using MapDraw software referring homoeologous chromosome locations of CS Ref Seq v1.0.

## Results

### Cytogenetic analysis of CS-*Ae*. *biuncialis* 2M^b^ disomic addition line TA7733

**Fig 1:**
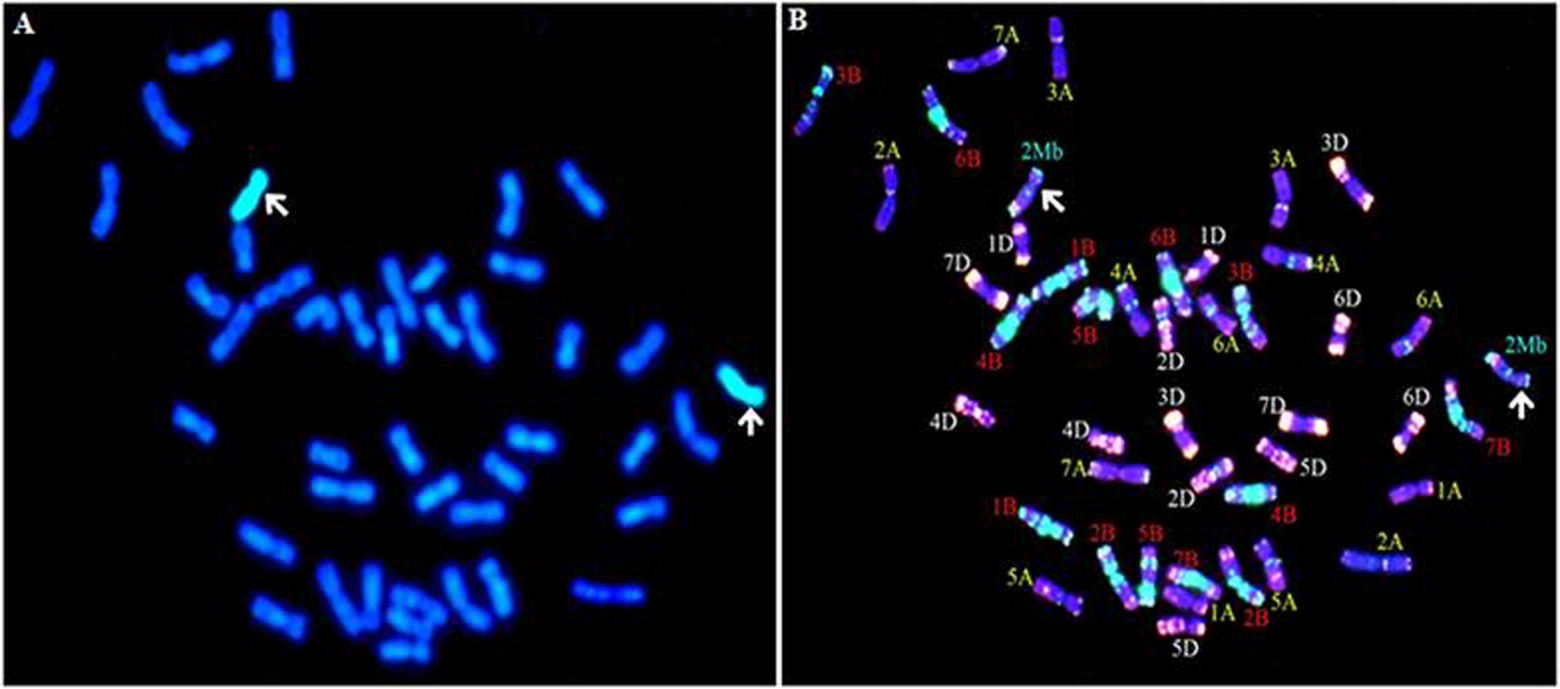
GISH and ND-FISH identification of CS-*Ae*. *biuncialis* 2M^b^ disomic addition line TA7733. (A) GISH patterns of CS-*Ae*. *biuncialis* 2M^b^ disomic addition line TA7733. Total gDNA of *Ae*. *comosa* was labelled with fluorescein-12-dUTP and visualized with green fluorescence. (B) ND-FISH patterns of CS-*Ae*. *biuncialis* 2M^b^ disomic addition line TA7733. Blue color indicated chromosomes counterstained with DAPI. Red color showed signals from oligos pAs1-1, pAs1-3, pAs1-4, pAs1-6, AFA-3 and AFA-4. Green color showed signals from oligos pSc119.2–1 and (GAA)_10_. The arrows indicated *Ae*. *biuncialis* chromosome 2M^b^.

GISH and ND-FISH were respectively performed to confirm the chromosome composition of CS-*Ae*. *biuncialis* 2M^b^ disomic addition line TA7733 by using fluorescein-labeled gDNA from M genome donor *Ae*. *comosa* as a probe and wheat CS DNA as blocker. As shown in Fig 1, there were 44 chromosomes including 42 wheat chromosomes and plus a pair of *Ae*. *biuncialis* 2M^b^ chromosomes in TA7733, confirming the disomic addition of chromosome 2M^b^.

### Assay of powdery mildew resistance of CS-*Ae*. *biuncialis* 2M^b^ disomic addition line TA7733

**Fig 2:**
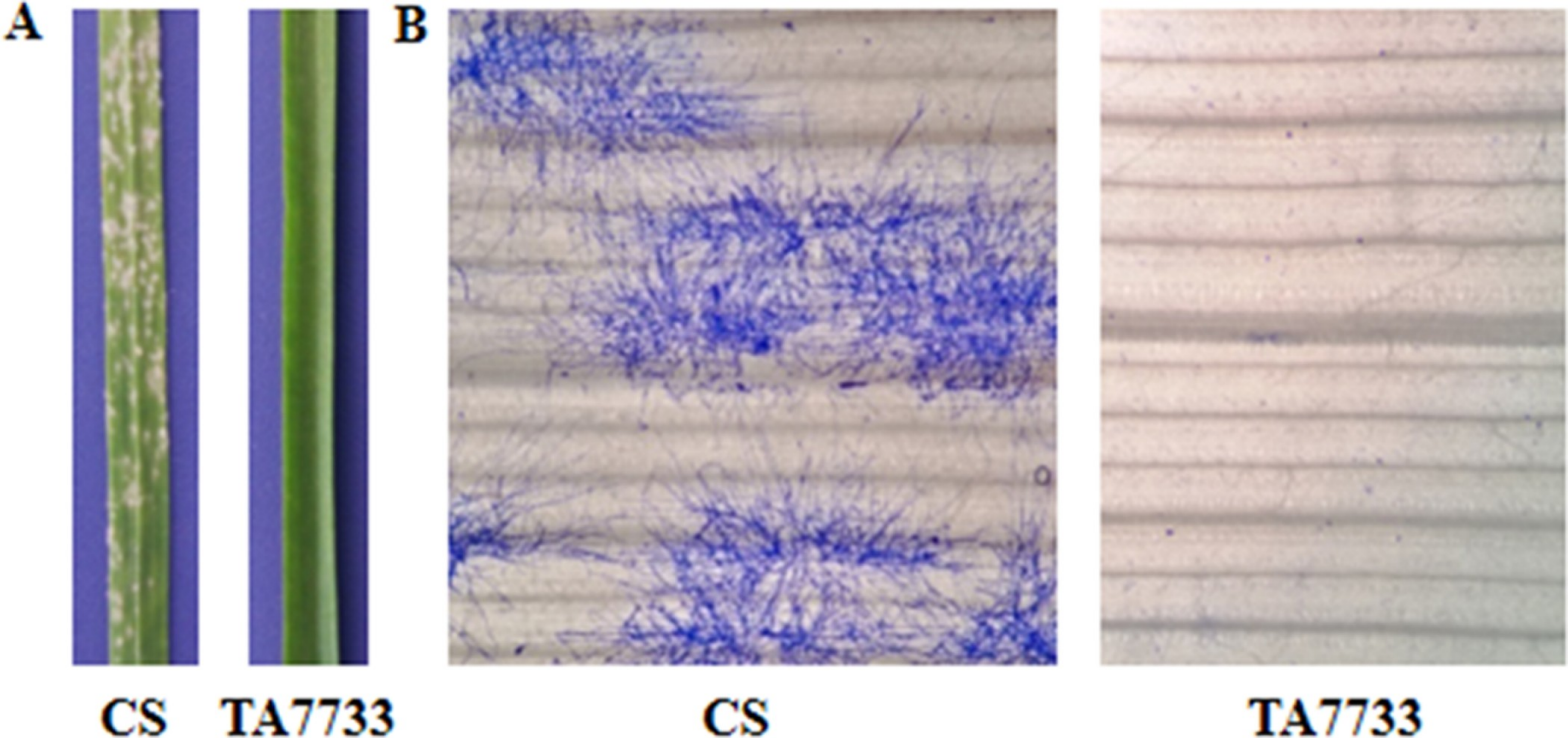
Powdery mildew resistance assay of CS-*Ae*. *biuncialis* 2M^b^ disomic addition line TA7733 and CS. (A) Disease symptoms of the first leaf of TA7733 and CS at 10 days post *Bgt* inoculation. (B) Microscopic observation of *Bgt* development on leaves of TA7733 and CS after staining with coomassie brilliant blue-R-250.

A mixture of prevalent *Bgt* isolates collected in Henan Province was used to inoculate seedlings with fully-extended first leaves of TA7733 and its recipient parent CS in the greenhouse. Ten days post-inoculation, the leaves of CS were covered with a large number of *Bgt* hyphae, with ITs of 3–4, whereas TA7733 showed only stunted spores, with ITs 0 to 1 (Fig 2A). Microscopic observation of first leaf segments stained with coomassie brilliant blue displayed that leaves of susceptible CS were covered with hyphae and spores had formed, while TA7733 only had a few blue spores on leaves (Fig 2B), further confirming that TA7733 was high resistance to powdery mildew. Since CS forms the genetic background TA7733 and is susceptible, the gene(s) conferring resistance to powdery mildew was therefore mapped to chromosome 2M^b^ derived from *Ae*. *biuncialis*.

The resistance spectrum of TA7733 was further assayed at the seedling stage by inoculation of 15 prevalent *Bgt* isolates collected from different regions of China. As shown in Table 1, CS-*Ae*. *biuncialis* 2M^b^ disomic addition line TA7733 showed high level of resistance (IT = 0 or 1) to all the 15 *Bgt* isolates tested, whereas its recipient parent CS was highly susceptible (IT = 4 or 3) to all tested *Bgt* isolates. These results indicated that the chromosome 2M^b^ in TA7733 conferred broad-spectrum resistance to powdery mildew of wheat.

**Table 1:** Infection types of CS-*Ae*. *biuncialis* 2M^b^ disomic addition line TA7733 and CS for different *Bgt* isolates at the seedling stage.

Isolates	Y01	Y02	Y03	Y04	Y05	Y06	Y07	Y08	Y09	Y10	Y11	Y14	Y15	Y17	Y18
**TA7733**	0	0	0	0	0	1	0	0	0	0	1	0	0	0	0
**CS**	4	4	3	4	4	4	4	4	4	4	4	4	4	4	3

0 as immune, 0; as nearly immune, 1 as highly resistant, 2 as moderately resistant, 3 as moderately susceptible, 4 as highly susceptible.

### Transcriptome sequencing, *de novo* assembly and functional annotation

**Fig 3:**
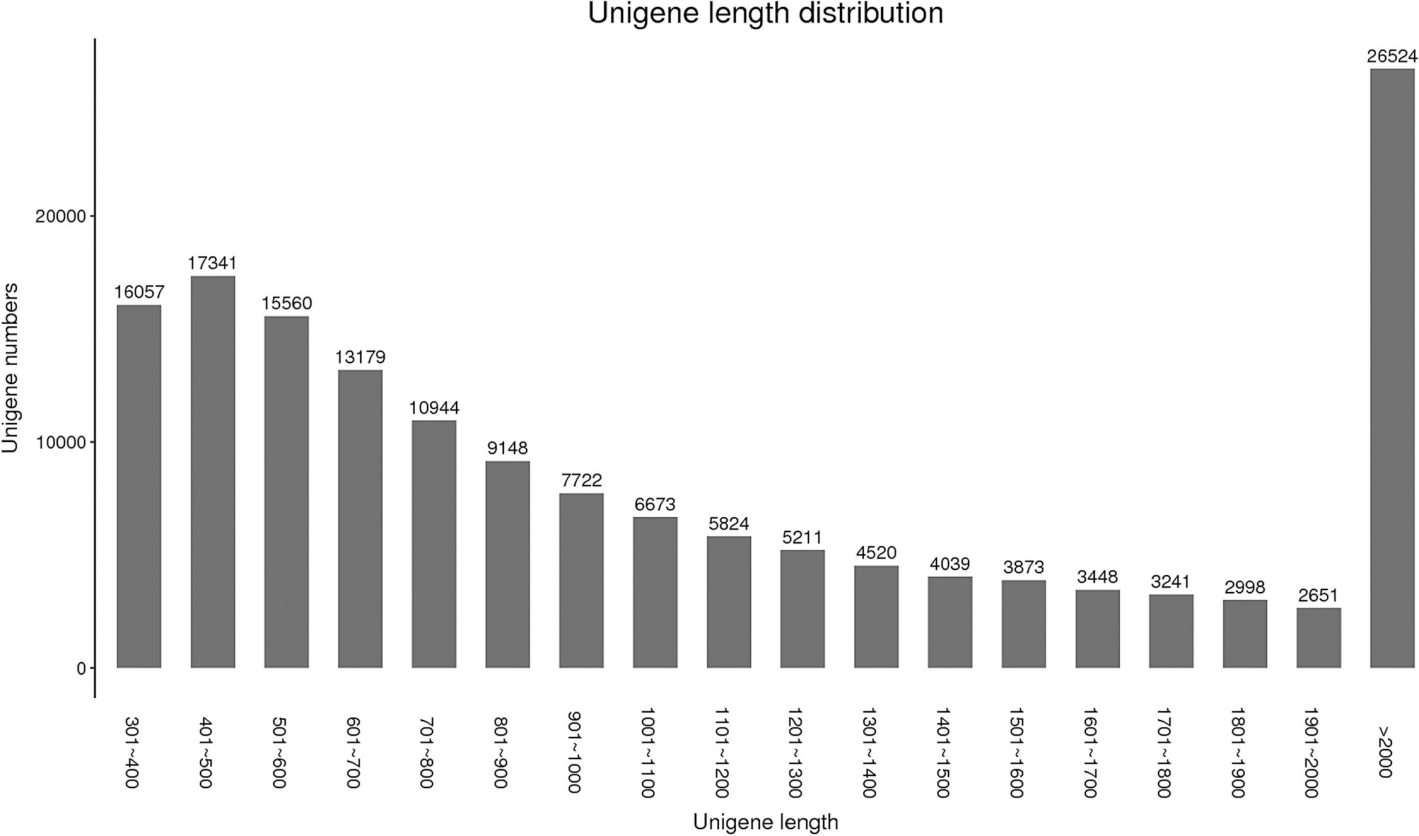
Length distribution of the assembled transcripts of CS-*Ae*. *biuncialis* 2M^b^ disomic addition line TA7733.

RNA-seq of CS-*Ae*. *biuncialis* 2M^b^ disomic addition line TA7733 and its recipient parent CS were respectively conducted pre and post *Bgt*-infection. A total of 158,953 unigenes were assembled with a total length of 198,364,757 bp. The average unigene size was 1247.95 bp ranging from 301 to 19,496 bp (Fig 3). Gene function annotation with Blastx to the six public databases (NCBI NR protein, SWISSPROT, GO, KOG, KEGG and PRG databases) using a cutoff E-value of 10^−5^ resulted in 86,196 (54.23%), 48,724 (30.65%), 40,543 (25.51%), 37,008 (23.28%), 13,414 (8.44%) and 10,969 (6.92%) annotated unigenes, respectively (Table 2). Of which, 86,862 (54.65%) unigenes matched to at least one of the databases.

**Table 2:** Functional annotation of the unigenes by transcriptome sequencing of TA7733.

database	NR	SWISSPROT	KOG	KEGG	GO	PRG	anno-union
**annotation numbers**	86,196	48,724	37,008	13,414	40,543	10,969	86,862
**annotation ratio (%)**	54.23	30.65	23.28	8.44	25.51	6.90	54.65

**Fig 4:**
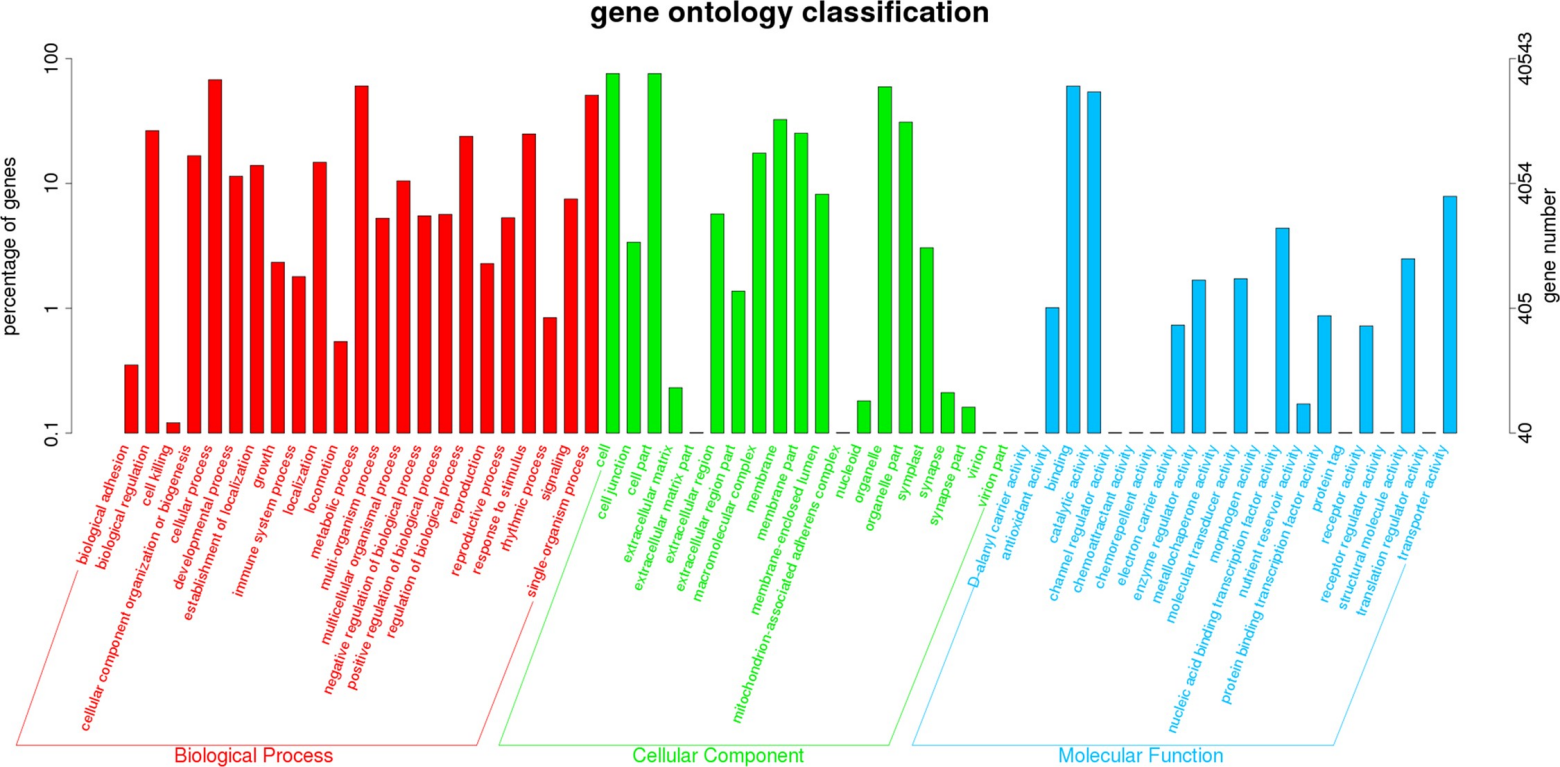
Histogram of GO categories of unigenes of CS-*Ae*. *biuncialis* 2M^b^ disomic addition line TA7733.

GO is an international classification system for standardized gene functions, which have three categories: biological process, molecular function and cellular component. A total of 40,543 (25.51% of 158,953) unigenes were assigned to one or more GO term annotations (Fig 4 and S1 Table), of which, “cellular process” (27,404; 67.59% of 40,543), “metabolic process” (24,470; 60.35% of 40,543), and “single-organism process” (20,606; 50.82% of 40,543) were the cardinal terms in the biological process category. In the cellular component category, “cell” (30,742; 75.82% of 40,543), “cell part” (30,695; 75.71% of 40,543), and “organelle” (24,100; 59.44% of 40,543) were the most abundant terms. “Binding” (24,379; 60.13% of 40,543) and “catalytic activity” (21,879; 53.96% of 40,543) were the most representative terms in the molecular function category. Instead, only a few unigenes assigned into the terms of “extracellular matrix part” (9; 0.02% of 40,543), “protein tag” (8; 0.02% of 40,543) and “receptor regulator activity” (1; 0.0024% of 40,543).

**Fig 5:**
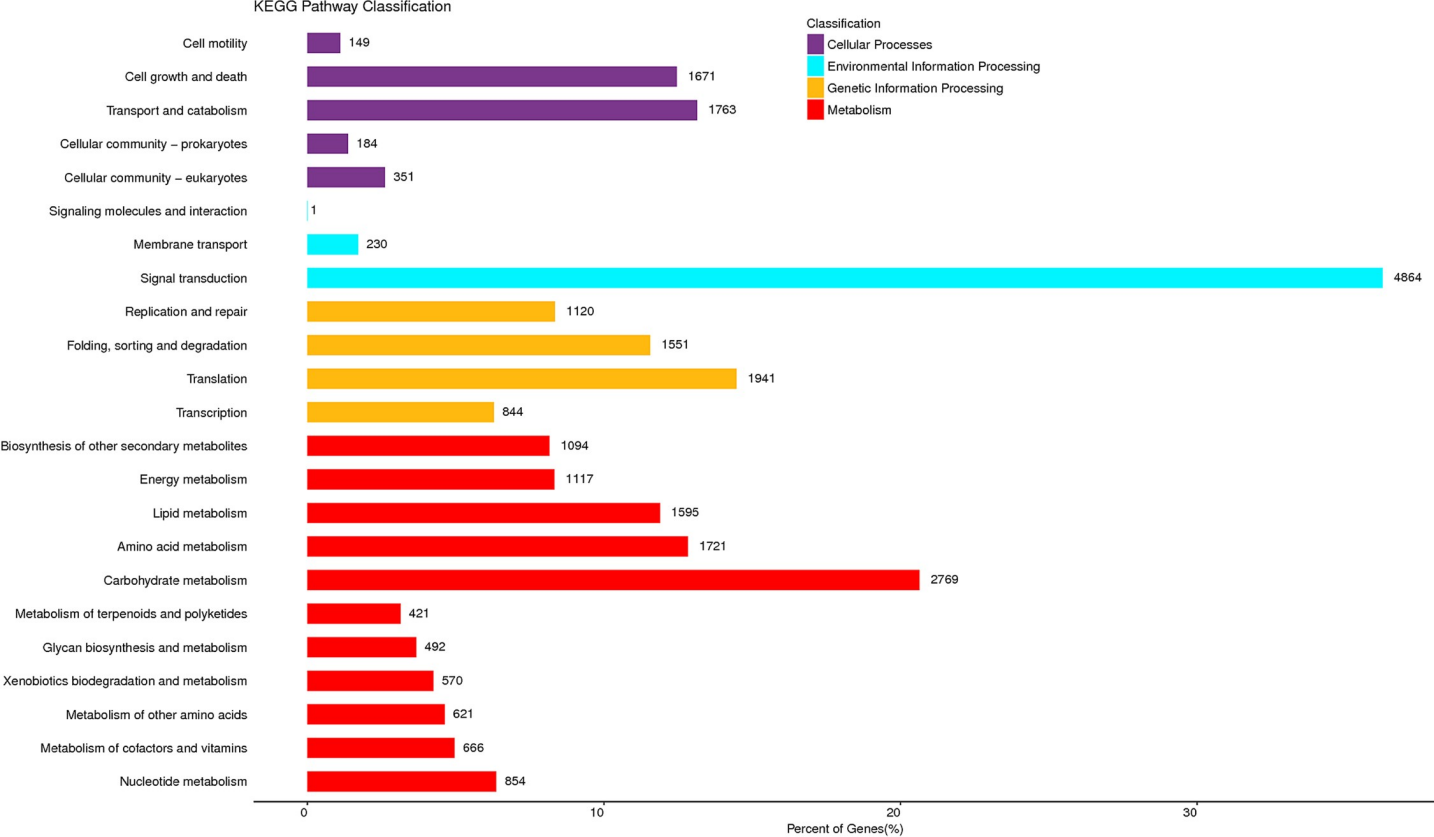
Clusters of KEGG functional classifications of unigenes of CS-*Ae*. *biuncialis* 2M^b^ disomic addition line TA7733.

The KEGG database was used to systematically describe the pathway where the unigenes involved. Out of a total 158,953 annotated unigenes, 26,589 unigenes were assigned to 23 KEGG pathways (Fig 5 and S2 Table). The most representative pathways in which unigenes involved were the metabolic pathways (11,920, 44.83%), genetic information processing (5,456, 20.52%), environmental information processing (5,095, 19.16%) and cellular processes (4,118, 15.49%).

### Analyses of genes involved in responses to *Bgt* infection from TA7733

One of the objectives of this study was to explore putative R genes specific to *Ae*. *biuncialis* chromosome 2M^b^, which should be only expressed in TA7733 other than in CS. Based on pairwise comparison of unigenes of TA7733 vs CS, a total of 7,278 genes were uniquely expressed in TA7733, of which 4,382 unigenes were significantly differentially expressed post vs before *Bgt*-inoculation, and the remaining 2,896 unigenes had insignificantly different expression levels. In consideration of the fact that expression levels of some cloned resistance genes did show no significant difference before and after pathogen infection [9,41], these 2,896 unigenes of TA7733 specificity were also taken for a further selection of chromosome 2M^b^-derived R-genes involved in powdery mildew resistance.

To analyze the biological pathways of these 4,382 unigenes, the statistical enrichment of differentially expressed genes (DEGs) in KEGG pathways were tested using the KOBAS software. In consequence, 399 out of 4,382 DEGs were allocated to 162 KEGG pathways (S3 Table). The most representative pathways included the phenylpropanoid biosynthesis (28, 7.02%), plant hormone signal transduction (23, 5.76%), flavonoid biosynthesis (15, 3.76%), stilbenoid, diarylheptanoid and gingerol biosynthesis (14, 3.51%) and MAPK signaling pathway-plant (14, 3.51%), then followed by glutathione metabolism (13, 3.26%), protein processing in endoplasmic reticulum (13, 3.26%), and metabolism of xenobiotics by cytochrome P450 (12, 3.00%). These annotations provided valuable clues in the investigation of the specific processes and identification of the genes involved in powdery mildew resistance conferred by *Ae*. *biuncialis* 2M^b^ chromosome.

### Screening and verification of disease resistance gene of chromosome 2M^b^ specificity

Based on transcriptome data analysis, 7,278 unigenes were uniquely expressed in TA7733. Only 295 of 7,278 unigenes were annotated as putative R-genes by Blastx alignment against the PRG and NCBI databases. However, of 295 R-genes sequences when blastn searched against CS Ref Seq v1.0, only 61 (20.68%) R-genes mapped to wheat homoeologous group 2, and the remaining 234 (79.32%) R-genes to none of the homoeologous group 2 chromosome of wheat.

In order to verify whether these 61 R genes which had an orthologous copy on wheat homoeologous group 2 were derived from *Ae*. *biuncialis* chromosome 2M^b^, a total of 61 sets of PCR primer pairs were designed based on transcriptome sequences of these R genes. PCR amplification of gDNA of CS and TA7733 confirmed 40 R genes to be specific to chromosome 2M^b^, which producing unique amplification in CS-*Ae*. *biuncialis* 2M^b^ disomic addition line TA7733 (S1 Fig and S4 Table). So these 40 R genes were considered as putative candidate genes involved in powdery mildew resistance conferred by *Ae*. *biuncialis* 2M^b^ chromosome.

Chromosome structure variation such as translocation occurs during biological evolution process, whether it occurred in wheat or its wild relatives, will lead to the changes of homoeologous groups. Among the remaining 234 putative R genes of TA7733 specificity which were mapped to none of the homoeologous group 2 chromosome, additional 13 R genes were verified to be also chromosome 2M^b^ specific by PCR analysis using primer pairs designed based on their transcriptome sequences (S1 Fig and S4 Table). These 13 R genes were also considered as candidate genes involved in powdery mildew resistance conferred by *Ae*. *biuncialis* 2M^b^ chromosome, which adding the number of candidate R genes of 2M^b^ specificity to a total of 53 (Tables 3 and S5 and S6).

**Table 3:** The functions of 53 disease resistance gene candidates from *Ae*. *biuncialis* chromosome 2M^b^.

Unigene IDs	Gene annotation	Expression regulation	Similarity to wheat homoeologous group
**CL84424Contig1**^**a**^	Cysteine-rich receptor-like protein kinase 26 [*Ae*. *tauschii*]	unregulated	83% (2B)
**CL93721Contig1**^**a**^	G-type lectin S-receptor-like serine/threonine-protein kinase At1g11300 [*Hordeum vulgare*]	unregulated	90% (2B)
**CL89447Contig1**^**b**^	LRR and NB-ARC domains-containing disease resistance protein (best arabidopsis hit); NBS-LRR disease resistance protein, putative, expressed (best rice hit) (CNL)	unregulated	82% (2A)
**CL90029Contig1**^**b**^	NBS-LRR disease resistance protein-like protein (NBS-LRR1) [*H*. *vulgare*] (CNL)	unregulated	88% (2B)
**CL88613Contig1**^**a**^	Predicted: disease resistance protein RGA2-like [*Brachypodium distachyon*]	unregulated	86% (2D)
**CL96221Contig1**^**a**^	Cysteine-rich receptor-like protein kinase 10 [*T*. *urartu*]	unregulated	79% (2B)
**CL106750Contig1**^**b**^	Putative disease resistance protein RGA1 [*Ae*. *tauschii*] (CNL)	unregulated	82% (2A)
**CL113949Contig1**^**a**^	Putative disease resistance protein RGA1 [*Ae*. *tauschii*]	unregulated	78% (2B)
**CL91742Contig1**^**b**^	(CNL)	unregulated	78% (2B)
**CL116612Contig1**^**b**^	Putative disease resistance RPP13-like protein 1 [*T*. *urartu*] (CNL)	unregulated	89% (2B)
**CL82670Contig1**^**a**^	Cytochrome P450 71D7 [*Ae*. *tauschii*]	unregulated	72% (2A)
**CL93169Contig1**^**b**^	NB-ARC domain-containing disease resistance protein (best arabidopsis hit); RGH1A, putative, expressed (best rice hit) (NL)	unregulated	79% (2B)
**CL108886Contig1**^**b**^	LRR and NB-ARC domains-containing disease resistance protein (best arabidopsis hit); NBS-LRR disease resistance protein, putative, expressed (best rice hit) (CNL)	unregulated	85% (2B)
**comp19533_c0_seq1_6**^**b**^	LRR and NB-ARC domains-containing disease resistance protein (best arabidopsis hit); NBS-LRR disease resistance protein, putative, expressed (best rice hit) (CNL)	unregulated	88% (2B)
**CL90483Contig1**^**b**^	Putative disease resistance RPP13-like protein 1 [*T*. *urartu*] (NL)	unregulated	83% (2A)
**CL85355Contig1**^**a**^	Disease resistance protein RPM1 [*T*. *urartu*]	unregulated	80% (2D)
**CL80063Contig1**^**b**^	Leucine-rich repeat protein kinase family protein (best arabidopsis hit) (RLP)	unregulated	80% (2D)
**CL66003Contig1**^**b**^	Putative disease resistance protein RGA4 [*Ae*. *tauschii*] (NL)	unregulated	76% (2B)
**CL119404Contig1**^**b**^	Putative disease resistance RPP13-like protein 1 [*Ae*. *tauschii*] (CNL)	unregulated	88% (2D)
**CL113652Contig1**^**b**^	Predicted: putative disease resistance RPP13-like protein 3 (LOC109731753) [*Ae*. *tauschii*] (NL)	unregulated	88% (2D)
**CL91022Contig1**^**a**^	Lectin-domain containing receptor kinase A4.3 [*Ae*. *tauschii*]	unregulated	78% (2B)
**CL85258Contig1**^**a**^	Predicted: G-type lectin S-receptor-like serine/threonine-protein kinase B120 [*Brachypodium distachyon*]	unregulated	77% (2B)
**CL105879Contig1**^**b**^	Putative LRR receptor-like serine/threonine-protein kinase [*Ae*. *tauschii*] (RLP)	unregulated	89% (2D)
**CL84846Contig1**^**a**^	Cysteine-rich receptor-like protein kinase 29 [*Ae*. *tauschii*]	unregulated	90% (2D)
**CL67241Contig1**^**b**^	(CNL)	unregulated	77% (2A)
**CL124Contig7**^**b**^	HOPZ-ACTIVATED RESISTANCE 1 (best arabidopsis hit); Leucine Rich Repeat family protein, expressed (best rice hit) (NL)	unregulated	85% (2A)
**CL89405Contig1**^**a**^	Putative disease resistance protein RGA4 [*Ae*. *tauschii*]	unregulated	86% (2A)
**CL119216Contig1**^**a**^	Putative serine/threonine-protein kinase receptor [*Ae*. *tauschii*]	unregulated	86% (2B)
**CL119539Contig1**^**b**^	(TNL)	unregulated	88% (2D)
**CL86521Contig1**^**a**^	Putative serine/threonine-protein kinase-like protein CCR3 [*Ae*. *tauschii*]	unregulated	83% (2D)
**CL29910Contig1**^**b**^	Disease resistance protein RGA2 [*Ae*. *tauschii*] (NL)	unregulated	68% (2A)
**CL87530Contig1**^**a**^	Wall-associated receptor kinase 4 [*T*. *urartu*]	unregulated	78% (2D)
**CL114224Contig1**^**b**^	NB-ARC domain-containing disease resistance protein (best arabidopsis hit); NB-ARC domain containing protein, expressed (best rice hit) (CNL)	unregulated	88% (2B)
**CL82700Contig1**^**a**^	Lectin-domain containing receptor kinase A4.3 [*Ae*. *tauschii*]	unregulated	80% (2B)
**comp84147_c0_seq1_6**^**b**^	TSA: *Triticum aestivum* cultivar Bobwhite isotig02189.flagleaf mRNA sequence (CNL)	up-regulated	88% (2D)
**CL92547Contig1**^**a**^	Predicted: probable LRR receptor-like serine/threonine-protein kinase At1g05700 (LOC109742478) [*Ae*. *tauschii*]	up-regulated	87% (2D)
**CL82789Contig1**^**a**^	Putative LRR receptor-like serine/threonine-protein kinase [*Ae*. *tauschii*]	up-regulated	77% (2A)
**CL88277Contig1**^**b**^	Predicted: probable leucine-rich repeat receptor-like protein kinase At1g35710 (LOC109774313) [*Ae*. *tauschii*] (RLP)	up-regulated	85% (2B)
**CL19981Contig2**^**a**^	Putative disease resistance protein RGA3 [*Ae*. *tauschii*]	down-regulated	80% (2B)
**CL75219Contig1**^**a**^	Predicted: putative disease resistance RPP13-like protein 3 (LOC109732887) [*Ae*. *tauschii*]	down-regulated	89% (2D)
**CL100654Contig1**^**b**^	NB-ARC domain-containing disease resistance protein (best arabidopsis hit) (NL)	unregulated	86% (6B)
**CL104996Contig1**^**b**^	(CNL)	unregulated	72% (7D)
**CL107524Contig1**^**b**^	(NL)	unregulated	80% (4A)
**CL107607Contig1**^**a**^	Disease resistance protein RGA2 [*Ae*. *tauschii*]	unregulated	82% (7B)
**CL465Contig5**^**b**^	*Hordeum vulgare* subsp. vulgare mRNA for predicted protein, complete cds, clone: NIASHv3099I02 (NL)	unregulated	73% (5B)
**CL66266Contig1**^**b**^	transmembrane receptors; ATP binding (best arabidopsis hit) (CNL)	unregulated	93% (6A)
**CL72629Contig1**^**b**^	(N)	unregulated	94% (4A)
**CL75868Contig1**^**b**^	TSA: *Triticum aestivum* cultivar Bobwhite isotig02316.flagleaf mRNA sequence (NL)	unregulated	81% (6B)
**CL86319Contig1**^**b**^	(NL)	unregulated	81% (6B)
**CL79458Contig1**^**b**^	*Hordeum vulgare* subsp. vulgare mRNA for predicted protein, complete cds, clone: NIASHv2142N02 (NL)	unregulated	86% (7D)
**comp121700_c0_seq1_5**^**b**^	(CNL)	unregulated	94% (3B)
**comp80277_c0_seq1_7**^**b**^	PEP1 receptor 1 (best arabidopsis hit); receptor-like protein kinase precursor, putative, expressed (best rice hit) (RLP)	unregulated	92% (4A)
**comp93868_c0_seq1_7**^**b**^	Leucine-rich repeat transmembrane protein kinase protein (best arabidopsis hit); senescence-induced receptor-like serine/threonine-protein kinase precursor, putative, expressed (best rice hit) (RLP)	unregulated	88% (3B)

^a^ indicated these R genes were assigned by alignment to NCBI database.

^b^ indicated these R genes were assigned by alignment to PRG database.

Alignment of these 53 R genes to PRG database assigned 33 putative genes, of which 14 R genes were in CNL class which contains a predicted coiled-coil (CC) structures, a central nucleotide-binding (NB) subdomain and a leucine-rich repeat (LRR) domain, 12 in NL class containing NBS and LRR domains, but lack of CC domain, five in class RLP which contains leucine-rich receptor-like repeat, a transmembrane region of 25AA, and a short cytoplasmic region, each one for TNL class which contains a central NB subdomain, a LRR domain, a interleukin-1 receptor (1L-1R) domain, and N class only containing NBS domain (Table 4). The remaining 20 putative R genes aligned to NCBI database, were predicted encoding protein kinase, disease resistance protein RGA, disease resistance protein RP and cytochrome P450, respectively.

**Table 4:** The types of 2M^b^-derived R genes annotated by alignment against the PRG database.

Types of R genes	Expression unchanged		Expression up-regulated		Total	
	number	percentage (%)	number	percentage (%)	number	percentage (%)
**CNL**	13	39.39	1	3.03	14	42.42
**NL**	12	36.36	0	0.00	12	36.36
**RLP**	4	12.12	1	3.03	5	15.15
**TNL**	1	3.03	0	0.00	1	3.03
**N**	1	3.03	0	0.00	1	3.03
**total**	31	93.94	2	6.06	33	100.00

CNL: contains a central nucleotide-binding (NB) subdomain, a leucine rich repeat (LRR) domain, and a predicted coiled-coil (CC) structures. NL: contains NBS and LRR domains, and lack of CC domain. RLP: contains leucine-rich receptor-like repeat, a transmembrane region of 25AA, and a short cytoplasmic region. TNL: contains a central NB subdomain, a LRR domain, and a interleukin-1 receptor (1L-1R) domain. N: contains NBS domain only, lack of LRR.

**Fig 6:**
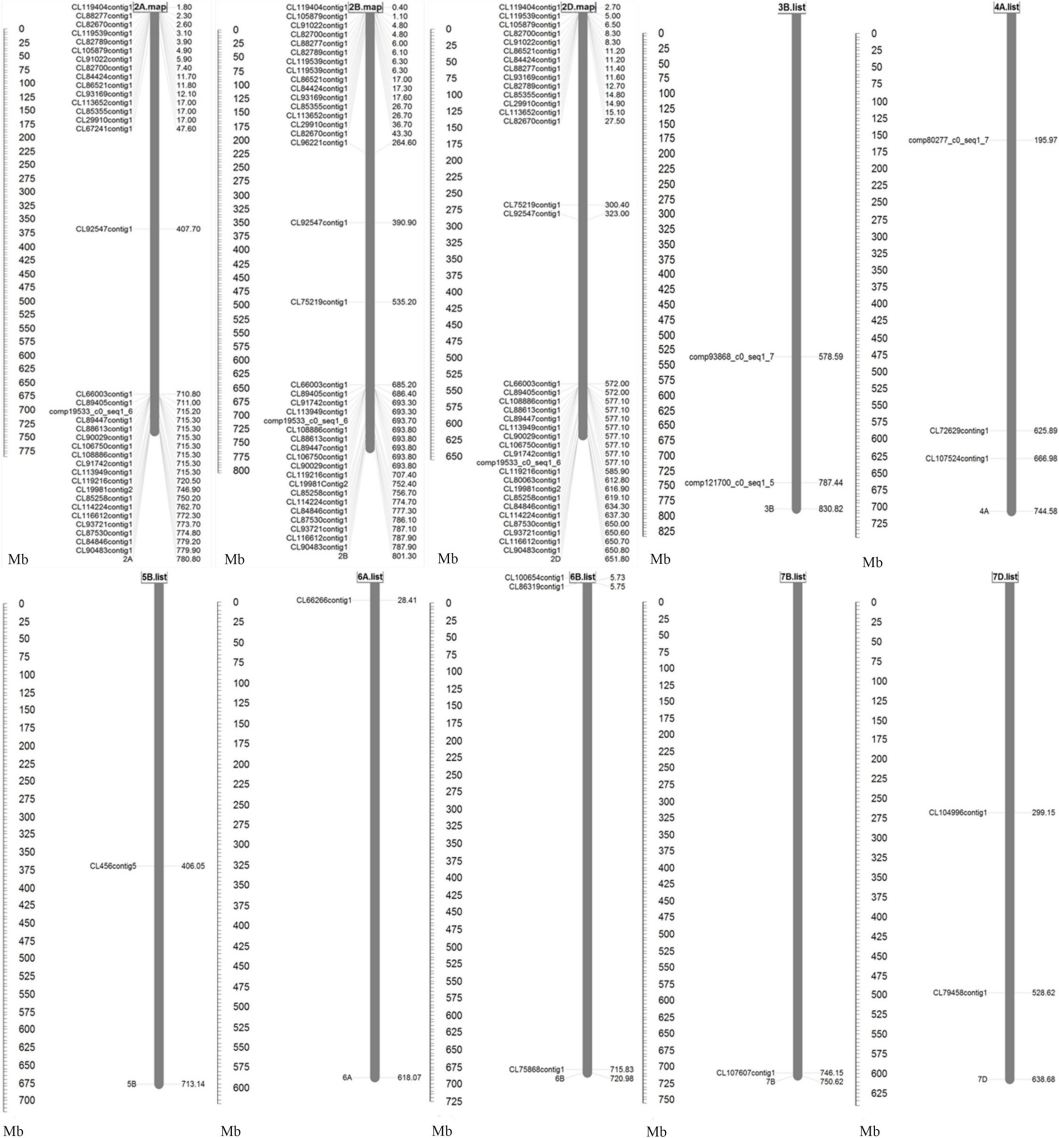
Comparative map of 53 R genes of 2M^b^ specificity based on alignment with CS Ref Seq v1.0.

Comparative mapping was carried out by using MapDraw software based on alignment of sequences of these 53 R genes of *Ae*. *biuncialis* chromosome 2M^b^ specificity with those in CS Ref Seq v1.0 (Fig 6). The maps showed that 21, 16 and three R genes were located to the terminal of the long arms and the short arms, and close to the centromeres of wheat homoeologous group 2 chromosomes, respectively. Whereas the remaining 13 R genes were mapped to none-homoeologous group 2, which included wheat chromosomes 3B, 4A, 5B, 6A, 6B, 7B and 7D (Fig 6).

## Discussion

Development of resistant wheat varieties is the most important and environment-friendly way to control *Bgt-*caused damages. The genes with broad spectrum and durability resistance make them highly valuable in wheat breeding programs [29]. Wild relatives of common wheat harbored considerable genetic diversity for powdery mildew resistance. For example, the wild relatives of common wheat, *Secale cereale*, *Dasypyrum villosum* and *Ae*. *searsii* conferred powdery mildew resistance gene *Pm7*, *PmJZHM2RL*, *Pm62* and *Pm57* from homoeologous group 2 [38, 42–44]. In this study, resistance assay by using 15 *Bgt* isolates collecting from different regions in China, verified that *Ae*. *biuncialis* 2M^b^ chromosome in TA7733 conferred broad-spectrum resistance to powdery mildew of wheat. Currently no any other catalogued *Pm* genes were reported to be derived from *Ae*. *biuncialis* homoeologous group 2. Therefore the resistance gene(s) on *Ae*. *biuncialis* 2M^b^ chromosome should be a new *Pm* gene(s).

Previous studies have generally focused on the significantly differentially expressed genes in interactions between plant and pathogens to explore disease resistance-related genes by transcriptome sequencing [45–47]. However, it was reported that expressions of some cloned genes of disease resistance in plants were not significantly up-regulated after pathogens infection. For example, Zou et al. (2017) reported that the transcription levels of *Pm60*, a map-based cloned powdery mildew gene, showed no significant differences at various time points after *Bgt* E09 infection based on qRT-PCR analysis [9]. Li et al. (2017) also discovered that the expression levels of broad-spectrum blast resistance gene *bsr-d1* in rice were not significantly up-regulated after blast infection [41]. So, the opportunity to discover disease resistance gene candidates might be undermined if only significantly regulated genes were chosen. In this study we explored *Bgt*-resistance related candidate genes from all specifically expressed R genes in TA7733 regardless of significance of their expression level changes post vs before *Bgt*-infection. After PCR verification by using R gene sequence-based primer sets and integrating transcriptome sequences blastn against CS Ref Seq v1.0, we finally verified 53 R genes candidates of chromosome 2M^b^ specificity, which included 47 unregulated, four up-regulated and two down-regulated genes.

Isolation of plant resistance (R) gene is greatly helpful to breed resistant varieties and elucidates resistance molecular mechanisms. Conventional map-based cloning proved to be a effective method to clone R genes [45], however, it is time consuming and difficult to fine map R genes from wild relatives of common wheat due to lack of exchange and recombination between the alien chromatin and wheat homoeologous counterpart. Of more than 70 R genes against diverse pathogens currently isolated from various plants by using map-based cloning [48], nearly three quarters of R genes encoded NBS-LRR protein, which reportedly recognized pathogens and initiated defense responses subsequently [48]. To date, five out of 89 *Pm* genes including *Pm2* [49], *Pm3* [50], *Pm8* [51], *Pm21* [28,52] and *Pm60* [9], have been cloned, all these genes encoded CC-NBS-LRR proteins. In this study, 14 out of 53 2M^b^-specific R genes were predicted to encode CC-NBS-LRR protein. These 14 R genes should be considered as the most promising candidate genes for further isolating and cloning *Pm* genes carried by *Ae*. *biuncialis* chromosome 2M^b^.

GISH is a popular visual method to identify alien chromosome or chromatin in wheat background. Whereas GISH is expensive and time-consuming especially used to screen a large population derived from distant crossing between wheat and its wild relatives [53]. In contrast, molecular markers are not affected by environmental conditions, tissue or developmental stage and gene expression, and possess high genetic polymorphism [54,55]. So the development and application of molecular markers have been considered as new and low-cost ways to quickly identify alien chromosomes or chromatin. High-throughput RNA-seq technology can generate large amounts of transcriptome sequences and has been widely used to develop molecular markers specific to chromosomes of wild relatives of wheat, especially those with limited genomic sequence references. For example, Li et al. (2017) developed 25 *D*. *villosum* 6V#4S-specifc markers using transcriptome data [56]. Wang et al. (2018) developed 134 *Ae*. *longissima* chromosome*-*specific markers by RNA-seq [30]. Li et al. (2019) developed 76 molecular markers specific to the chromosome 1V to 7V of *D*. *villosum*#4 based on transcriptome data [31]. Furthermore, the transcription sequences were highly conserved and might be associated with the genes that related to a definite trait [57]. Therefore, the markers developed by transcriptome sequencing will accelerate the identification of candidate functional genes, and increase the efficiency of marker-assisted selection [57,58]. In this study, 53 functional molecular markers of R genes based on transcriptome data analyses were verified to be specific to *Ae*. *biuncialis* chromosome 2M^b^. These markers will be useful to assist the transfer resistance gene(s) from 2M^b^ into common wheat by inducing CS-*Ae*. *biuncialis* 2M^b^ homoeologous recombination for wheat disease breeding in the future.

## Conclusions

In summary, powdery mildew resistance gene(s) on *Ae*. *biuncialis* chromosome 2M^b^ was verified to be board-spectrum in this study. It could be a valuable disease-resistance resource for wheat breeding programs. Fifty-three disease resistance gene candidates of 2M^b^ specificity, which were selected based on transcriptome sequencing analyses, will be greatly helpful to further isolate and clone *Pm* gene(s) derived from chromosome 2M^b^ and provide the insights into molecular mechanism of 2M^b^-conferred powdery mildew resistance. Furthermore, 53 R gene sequence-based functional molecular markers of 2M^b^ specificity in this study will facilitate the transfer of resistance gene(s) from 2M^b^ to common wheat by inducing CS-*Ae*. *biuncialis* homoeologous recombination.

## Supporting information

S1 Fig Amplification patterns of 53 candidate *Ae*. *biuncialis* chromosome 2M^b^-specific primers. (M) 100 bp DNA Ladder. (1, 3) CS. (2, 4) TA7733. (A) CL119404Contig1. (B) CL88277Contig1. (C) CL82670Contig1. (D) 82789Contig1. (E) CL82700Contig1. (F) CL85355Contig1. (G) CL66003Contig1. (H) CL89405Contig1. (I) CL106750Contig1. (J) CL119216Contig1. (K) CL19981Contig2. (L) CL93721Contig1. (M) CL84424Contig1. (N) CL88613Contig1. (O) CL91022Contig1. (P) 96221Contig1. (Q) comp19533_c0_seq1_6. (R) CL85258Contig1. (S) CL113949Contig1. (T) CL86521Contig1. (U) CL105879Contig1. (V) CL90029Contig1. (W) CL84846Contig2. (X) CL87530Contig1. (Y) CL29910Contig1. (Z) CL92547Contig1. (AA) CL75219Contig1. (AB) CL108886Contig1. (AC) CL113652Contig1. (AD) CL80063Contig1. (AE) CL89447Contig1. (AF) CL93169Contig1. (AG) CL114224Contig1. (AH) CL116612Contig1. (AI) CL67241Contig1. (AJ) CL119539Contig1. (AK) CL90483Contig1. (AL) CL91742Contig1. (AM) comp84147_c0_seq1_6. (AN) CL124Contig7. (AO) CL100654Contig1. (AP) CL104996Contig1. (AQ) CL107524Contig1. (AR) CL107607Contig1. (AS) CL465Contig5. (AT) CL66266Contig1. (AU) CL72629Contig1. (AV) CL75868Contig1. (AW) CL86319Contig1. (AX) CL79458Contig1. (AY) comp121700_c0_seq1_5. (AZ) comp80277_c0_seq1_7. (BA) comp93868_c0_seq1_7.
(TIF)

S1 Raw_image (original image of S1 Fig): Raw images of amplification patterns of 53 candidate *Ae*. *biuncialis* chromosome 2M^b^-specific primers. Lanes: M, 100 bp Ladder DNA Marker; 1, common wheat CS; 2, CS-*Aegilops biuncialis* 2M^b^ disomic addition line TA77333; 3, common wheat CS; 4, CS-*Ae*. *biuncialis* 2M^b^ disomic addition line TA77333. (A) CL119404Contig1. (B) CL88277Contig1. (C) CL82670Contig1. (D) 82789Contig1. (E) CL82700Contig1. (F) CL85355Contig1. (G) CL66003Contig1. (H) CL89405Contig1. (I) CL106750Contig1. (J) CL119216Contig1. (K) CL19981Contig2. (L) CL93721Contig1. (M) CL84424Contig1. (N) CL88613Contig1. (O) CL91022Contig1. (P) 96221Contig1. (Q) comp19533_c0_seq1_6. (R) CL85258Contig1. (S) CL113949Contig1. (T) CL86521Contig1. (U) CL105879Contig1. (V) CL90029Contig1. (W) CL84846Contig2. (X) CL87530Contig1. (Y) CL29910Contig1. (Z) CL92547Contig1. (AA) CL75219Contig1. (AB) CL108886Contig1. (AC) CL113652Contig1. (AD) CL80063Contig1. (AE) CL89447Contig1. (AF) CL93169Contig1. (AG) CL114224Contig1. (AH) CL116612Contig1. (AI) CL67241Contig1. (AJ) CL119539Contig1. (AK) CL90483Contig1. (AL) CL91742Contig1. (AM) comp84147_c0_seq1_6. (AN) CL124Contig7. (AO) CL100654Contig1. (AP) CL104996Contig1. (AQ) CL107524Contig1. (AR) CL107607Contig1. (AS) CL465Contig5. (AT) CL66266Contig1. (AU) CL72629Contig1. (AV) CL75868Contig1. (AW) CL86319Contig1. (AX) CL79458Contig1. (AY) comp121700_c0_seq1_5. (AZ) comp80277_c0_seq1_7. (BA) comp93868_c0_seq1_7.
(PDF)

S1 Table Gene ontology of transcriptome of CS-*Ae*. *biuncialis* 2M^b^ disomic addition line TA7733. (XLS)

S2 Table The KEGG pathway classification of transcriptome of CS-*Ae*. *biuncialis* 2M^b^ disomic addition line TA7733. (XLS)

S3 Table KEGG pathway classification of DEGs of CS-*Ae*. *biuncialis* 2M^b^ disomic addition line TA7733. (XLS)

S4 Table The 53 *Ae*. *biuncialis* chromosome 2M^b^-specific markers developed in this study based on unigenes annotated as R genes. (XLS)

S5 Table The expression levels of 53 candidate disease resistance genes from *Ae*. *biuncialis* chromosome 2M^b^. (XLS)

S6 Table The list sequences of 53 candidate disease resistance genes from *Ae*. *biuncialis* chromosome 2M^b^. (XLS)
