# Supplementary figures and images for: Discovery of powdery mildew resistance gene candidates from Aegilops biuncialis chromosome 2Mb based on transcriptome sequencing
Source: PLoS One. 2019 Nov 11;14(11):e0220089. doi: 10.1371/journal.pone.0220089 (PMC6844473; doi:10.1371/journal.pone.0220089)

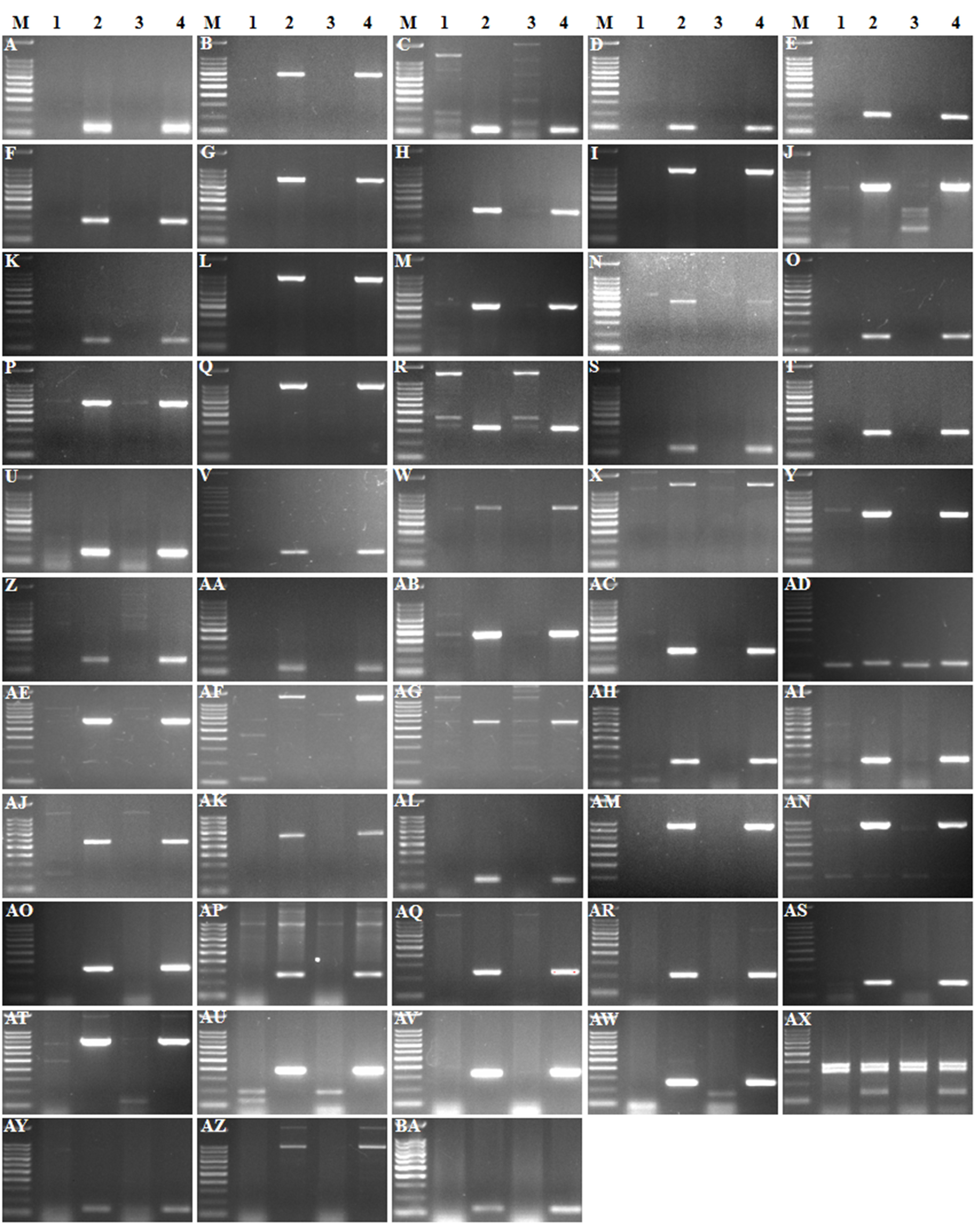

Supplement: S1 Fig — (M) 100 bp DNA Ladder. (1, 3) CS. (2, 4) TA7733. (A) CL119404Contig1. (B) CL88277Contig1. (C) CL82670Contig1. (D) 82789Contig1. (E) CL82700Contig1. (F) CL85355Contig1. (G) CL66003Contig1. (H) CL89405Contig1. (I) CL106750Contig1. (J) CL119216Contig1. (K) CL19981Contig2. (L) CL93721Contig1. (M) CL84424Contig1. (N) CL88613Contig1. (O) CL91022Contig1. (P) 96221Contig1. (Q) comp19533_c0_seq1_6. (R) CL85258Contig1. (S) CL113949Contig1. (T) CL86521Contig1. (U) CL105879Contig1. (V) CL90029Contig1. (W) CL84846Contig2. (X) CL87530Contig1. (Y) CL29910Contig1. (Z) CL92547Contig1. (AA) CL75219Contig1. (AB) CL108886Contig1. (AC) CL113652Contig1. (AD) CL80063Contig1. (AE) CL89447Contig1. (AF) CL93169Contig1. (AG) CL114224Contig1. (AH) CL116612Contig1. (AI) CL67241Contig1. (AJ) CL119539Contig1. (AK) CL90483Contig1. (AL) CL91742Contig1. (AM) comp84147_c0_seq1_6. (AN) CL124Contig7. (AO) CL100654Contig1. (AP) CL104996Contig1. (AQ) CL107524Contig1. (AR) CL107607Contig1. (AS) CL465Contig5. (AT) CL66266Contig1. (AU) CL72629Contig1. (AV) CL75868Contig1. (AW) CL86319Contig1. (AX) CL79458Contig1. (AY) comp121700_c0_seq1_5. (AZ) comp80277_c0_seq1_7. (BA) comp93868_c0_seq1_7. (TIF) [file pone.0220089.s001.tif]
