# Supplementary material for: Discovery of powdery mildew resistance gene candidates from Aegilops biuncialis chromosome 2Mb based on transcriptome sequencing
Source: PLoS One. 2019 Nov 11;14(11):e0220089. doi: 10.1371/journal.pone.0220089 (PMC6844473; doi:10.1371/journal.pone.0220089)

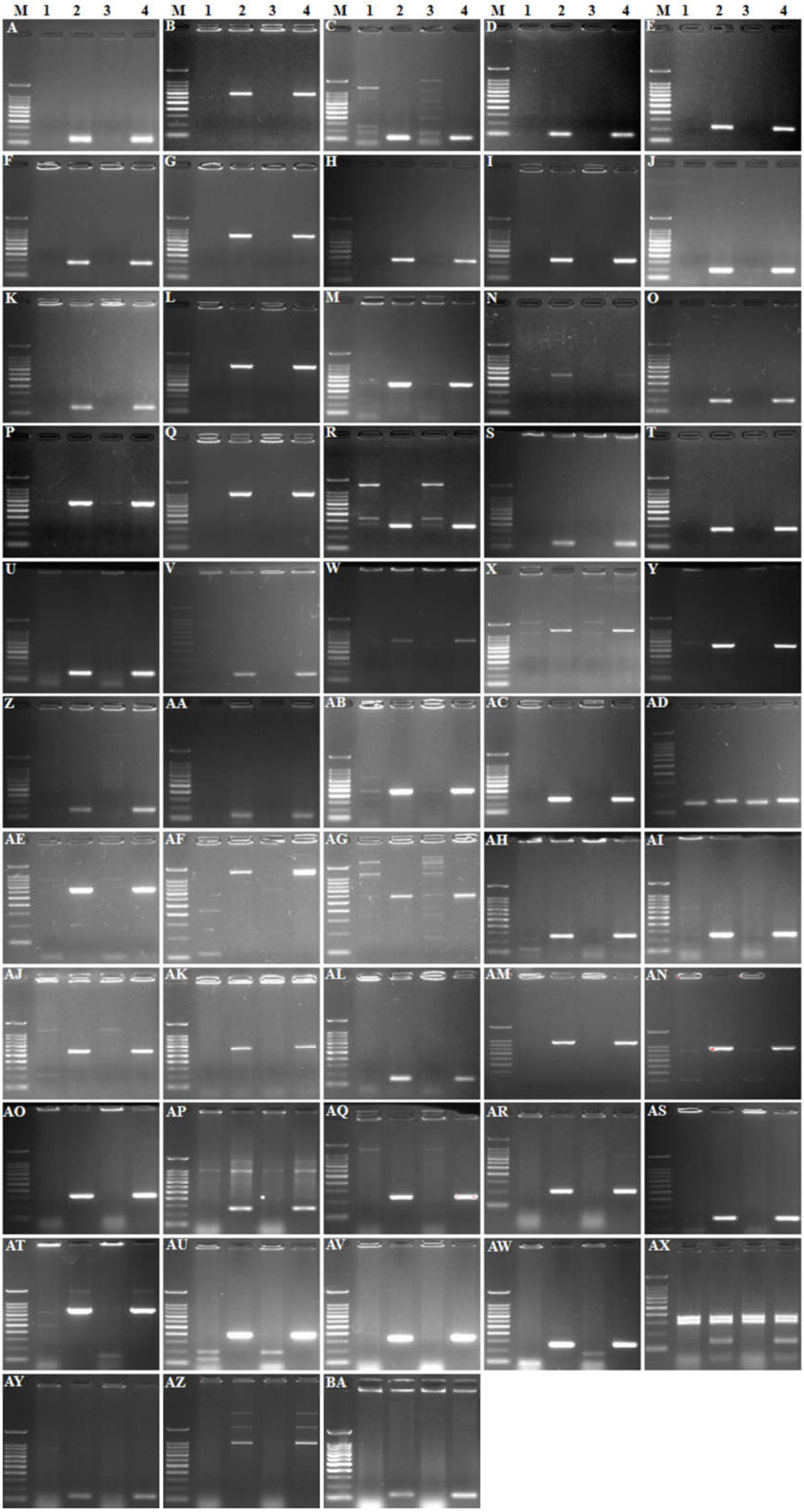
M 1 2 3 4 M 1 2 3 4 M 1 2 3 4 M 1 2 3 4 M 1 2 3 4
A
B
C
D
E
F
G
H
I
J
K
L
M
N
O
P
Q
R
S
T
U
V
W
X
Y
Z
AA
AB
AC
AD
AE
AF
AG
AH
AI
AJ
AK
AL
AM
AN
AO
AP
AQ
AR
AS
AT
AU
AV
AW
AX
AY
AZ
BA

S1_Raw_image (original image of S1 Fig): Raw images of amplification patterns of 53 candidate *Ae. biuncialis* chromosome $2M^b$-specific primers. Lanes: M, 100 bp Ladder DNA Marker; 1, common wheat CS; 2, CS-*Aegilops biuncialis* $2M^b$ disomic addition line TA77333; 3, common wheat CS; 4, CS-*Ae. biuncialis* $2M^b$ disomic addition line TA77333. (A) CL119404Contig1. (B) CL88277Contig1. (C) CL82670Contig1. (D) 82789Contig1. (E) CL82700Contig1. (F) CL85355Contig1. (G) CL66003Contig1. (H) CL89405Contig1. (I) CL106750Contig1. (J) CL119216Contig1. (K) CL19981Contig2. (L) CL93721Contig1. (M) CL84424Contig1. (N) CL88613Contig1. (O) CL91022Contig1. (P) 96221Contig1. (Q) comp19533_c0_seq1_6. (R) CL85258Contig1. (S) CL113949Contig1. (T) CL86521Contig1. (U) CL105879Contig1. (V) CL90029Contig1. (W) CL84846Contig2. (X) CL87530Contig1. (Y) CL29910Contig1. (Z) CL92547Contig1. (AA) CL75219Contig1. (AB) CL108886Contig1. (AC) CL113652Contig1. (AD) CL80063Contig1. (AE) CL89447Contig1. (AF) CL93169Contig1. (AG) CL114224Contig1. (AH) CL116612Contig1. (AI) CL67241Contig1. (AJ) CL119539Contig1. (AK) CL90483Contig1. (AL) CL91742Contig1. (AM) comp84147_c0_seq1_6. (AN) CL124Contig7. (AO) CL100654Contig1. (AP) CL104996Contig1. (AQ) CL107524Contig1. (AR) CL107607Contig1. (AS) CL465Contig5. (AT) CL66266Contig1. (AU) CL72629Contig1. (AV) CL75868Contig1. (AW) CL86319Contig1. (AX) CL79458Contig1. (AY) comp121700_c0_seq1_5. (AZ) comp80277_c0_seq1_7. (BA) comp93868_c0_seq1_7.

Methods

PCR amplification were conducted in 15 μl reaction volumes containing 2 μl template gDNA (100 ng/μl), 0.25 μl forward primer (10 μmol/l), 0.25 μl reverse primer (10 μmol/l), 7.5 μl Taq MasterMix (CW Bio Inc., Beijing, China) and 5 μl ddH_2O. PCR cycling conditions were as follows: 94°C for 5 min followed by 35 cycles of 94°C for 30 s, 50-66°C (depending on the Tm of the primers) for 30 s, and 72°C for 1 min, followed by a final 10-min extension at 72°C. The PCR products were digested with

four base-restriction enzymes. Five microliters of a restriction enzyme mixture containing 2.8 μl of ddH_2O, 2.0 μl of CutSmart buffer, and 0.2 μl of an enzyme stock solution was added to 15 μl of PCR products and incubated for 3.5 h at 65°C. The PCR or restricted PCR products were separated on a 2.0% agarose gel, stained with ethidium bromide, and visualized by Tanon 2500 Gel Imaging System (Tanon Science & Technology Co., Ltd., Shanghai, China).

M: 100 bp Ladder DNA Marker

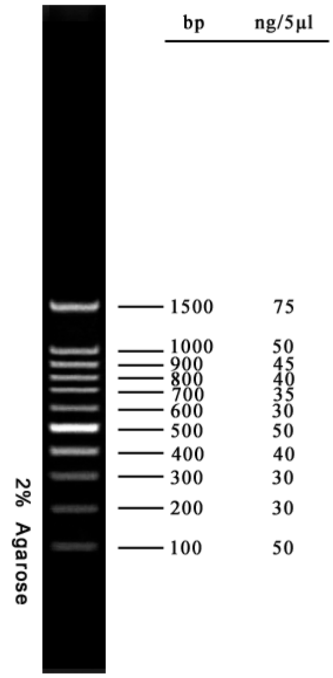

Supplement: S1 Raw_image — Lanes: M, 100 bp Ladder DNA Marker; 1, common wheat CS; 2, CS-Aegilops biuncialis 2Mb disomic addition line TA77333; 3, common wheat CS; 4, CS-Ae. biuncialis 2Mb disomic addition line TA77333. (A) CL119404Contig1. (B) CL88277Contig1. (C) CL82670Contig1. (D) 82789Contig1. (E) CL82700Contig1. (F) CL85355Contig1. (G) CL66003Contig1. (H) CL89405Contig1. (I) CL106750Contig1. (J) CL119216Contig1. (K) CL19981Contig2. (L) CL93721Contig1. (M) CL84424Contig1. (N) CL88613Contig1. (O) CL91022Contig1. (P) 96221Contig1. (Q) comp19533_c0_seq1_6. (R) CL85258Contig1. (S) CL113949Contig1. (T) CL86521Contig1. (U) CL105879Contig1. (V) CL90029Contig1. (W) CL84846Contig2. (X) CL87530Contig1. (Y) CL29910Contig1. (Z) CL92547Contig1. (AA) CL75219Contig1. (AB) CL108886Contig1. (AC) CL113652Contig1. (AD) CL80063Contig1. (AE) CL89447Contig1. (AF) CL93169Contig1. (AG) CL114224Contig1. (AH) CL116612Contig1. (AI) CL67241Contig1. (AJ) CL119539Contig1. (AK) CL90483Contig1. (AL) CL91742Contig1. (AM) comp84147_c0_seq1_6. (AN) CL124Contig7. (AO) CL100654Contig1. (AP) CL104996Contig1. (AQ) CL107524Contig1. (AR) CL107607Contig1. (AS) CL465Contig5. (AT) CL66266Contig1. (AU) CL72629Contig1. (AV) CL75868Contig1. (AW) CL86319Contig1. (AX) CL79458Contig1. (AY) comp121700_c0_seq1_5. (AZ) comp80277_c0_seq1_7. (BA) comp93868_c0_seq1_7. (PDF) [file pone.0220089.s002.pdf]
